# Differential Effect of Cobalt and Chromium Ions as Well as CoCr Particles on the Expression of Osteogenic Markers and Osteoblast Function

**DOI:** 10.3390/ijms19103034

**Published:** 2018-10-05

**Authors:** Andreas Drynda, Susanne Drynda, Jörn Kekow, Christoph Hubertus Lohmann, Jessica Bertrand

**Affiliations:** 1 Department of Orthopaedic Surgery, Otto-von-Guericke University, Leipziger Straße 44, D-39120 Magdeburg, Germany; andreas.drynda@med.ovgu.de (A.D.); susanne.drynda@med.ovgu.de (S.D.); christoph.lohmann@med.ovgu.de (C.H.L.); 2 Clinic for Rheumatology, Otto-von-Guericke University, Leipziger Straße 44, D-39120 Magdeburg, Germany; joern.kekow@med.ovgu.de

**Keywords:** TGF-beta, osteoblast, heavy metal ions, collagen, cell migration

## Abstract

The balance of bone formation and resorption is the result of a regulated crosstalk between osteoblasts, osteoclasts, and osteocytes. Inflammation, mechanical load, and external stimuli modulate this system. Exposure of bone cells to metal ions or wear particles are thought to cause osteolysis via activation of osteoclasts and inhibition of osteoblast activity. Co^2+^ ions have been shown to impair osteoblast function and the expression of the three transforming growth factor (TGF)-β isoforms. The current study was performed to analyze how Co^2+^ and Cr^3+^ influence the expression, proliferation, and migration profile of osteoblast-like cells. The influence of Co^2+^, Cr^3+^, and CoCr particles on gene expression was analyzed using an osteogenesis PCR Array. The expression of different members of the TGF-β signaling cascade were down-regulated by Co^2+^, as well as several TGF-β regulated collagens, however, Cr^3+^ had no effect. CoCr particles partially affected similar genes as the Co^2+^treatment. Total collagen production of Co^2+^ treated osteoblasts was reduced, which can be explained by the reduced expression levels of various collagens. While proliferation of MG63 cells appears unaffected by Co^2+^, the migration capacity was impaired. Our data may improve the knowledge of changes in gene expression patterns, and the proliferation and migration effects caused by artificial materials.

## 1. Introduction

Total hip arthroplasty (THA) is an effective and safe technique to treat degenerative, post-traumatic, and other diseases of the hip joint. One of the main reasons for a limited lifespan of THAs is aseptic loosening due to increased bone resorption [1,2]. The tight regulation of bone formation and resorption is therefore of great importance to ensure the THA function. Osteoblast activity is important during implant ingrowth and the prevention of implant loosening, which requires mature osteoblasts to deposit bone with remarkable spatial precision.

Many metal implant devices used in orthopaedic surgery are manufactured from cobalt and chromium alloys. Metal particles can be degraded into their respective ions in periprosthetic tissue [3,4,5]. Abrasive wear particles liberated from the articulation surfaces or cone-taper connections of metal-containing implants can induce adverse biological reactions in periprosthetic tissue as well as systemic effects caused by released metal ions (Co^2+^/Cr^3+^) and metal degradation products (e.g., oxides, insoluble metal salts, and metal-protein complexes) [6,7,8]. Particles and metal ions affect the cells of the surrounding tissues, including immune cells (lymphocytes, macrophages), bone cells (osteoblasts, osteoclasts), and fibroblasts [9]. These abrasive wear particles are thought to induce osteolysis by shifting the sensitive balance of bone homeostasis towards bone resorption or reduced osteoblast activity. The exact mechanisms of this interaction are only partially understood.

Bone homeostasis is tightly regulated by growth factors, cytokines, hormones, mechanical load, and other variables. Among these factors, transforming growth factor (TGF)-β and bone marrow proteins (BMPs) play an important role. BMP morphogen gradients regulate bone formation during embryogenesis [10]. Disruptions of TGF-β/BMP signaling have been implicated in multiple bone diseases including tumor metastasis [11]. It is known that TGF-β activates COL1A1 synthesis thereby providing the matrix for mineralization and bone formation [12,13]. Bone tissue is mainly composed of type I collagen, which is also thought to be a marker for osteoblast activity [14]. However, other collagens are also expressed in bone tissue to a smaller amount contributing either to the mechanical properties of the bone or regulating bioavailability of morphogens and cytokines. One of these collagens is type III collagen (Col3), which can be found in heterotypic fibrils with type I collagen (Col1) [15,16,17]. Other non-fibrilar collagens are also found in bone tissue, e.g., collagen XIV, which is found mainly in tissues containing type I collagen [18], and collagen XV which participates in extracellular matrix (ECM) organization in the early-phases of the osteogenic process and that is a prerequisite to promote the subsequent deposition of the mineral matrix [19]. The osteoblast, as the bone forming and remodelling cell, produces a variety of cytokines and chemokines, both under physiologic and pathological conditions regulating the osteoblast migration to the exact site of bone formation [20,21]. Alrabeah and co-workers demonstrated that Co^2+^ metal ions in the culture media induced the production of pro-inflammatory cytokines in human osteoblastic cells, thereby inducing a shift of bone homeostasis towards bone resorption [22]. Furthermore, it is known that Co^2+^ ions reduce the expression of all three TGF-β isoforms in osteosarcoma cell lines MG63 and SaOs-2 in a dose-dependent manner, with the strongest impact on TGF-β2 resulting in a change of the TGF-β isoform pattern [23]. It is still unknown whether these changes in the expression pattern of TGF-β isoforms are involved in the dysregulation of the periprosthetic bone metabolism and function of osteoblasts.

It was the aim of the study to investigate the effect of Co^2+^ and Cr^3+^ ions on signaling pathways of bone metabolism and osteoblast functions. We hypothesized that Co^2+^ and Cr^3+^ ions have different influence on cell migration and the expression of osteoblast related genes that may induce bone loss. Therefore, we used the osteoblast-like cell line MG63, stimulated these cells with Co^2+^ and Cr^3+^ ions (0–250 µM) as well as CoCr particles, and analyzed the migration properties and gene expression on a low density Profiler PCR Array.

## 2. Results

### 2.1. Cell Proliferation

**Figure 1:**
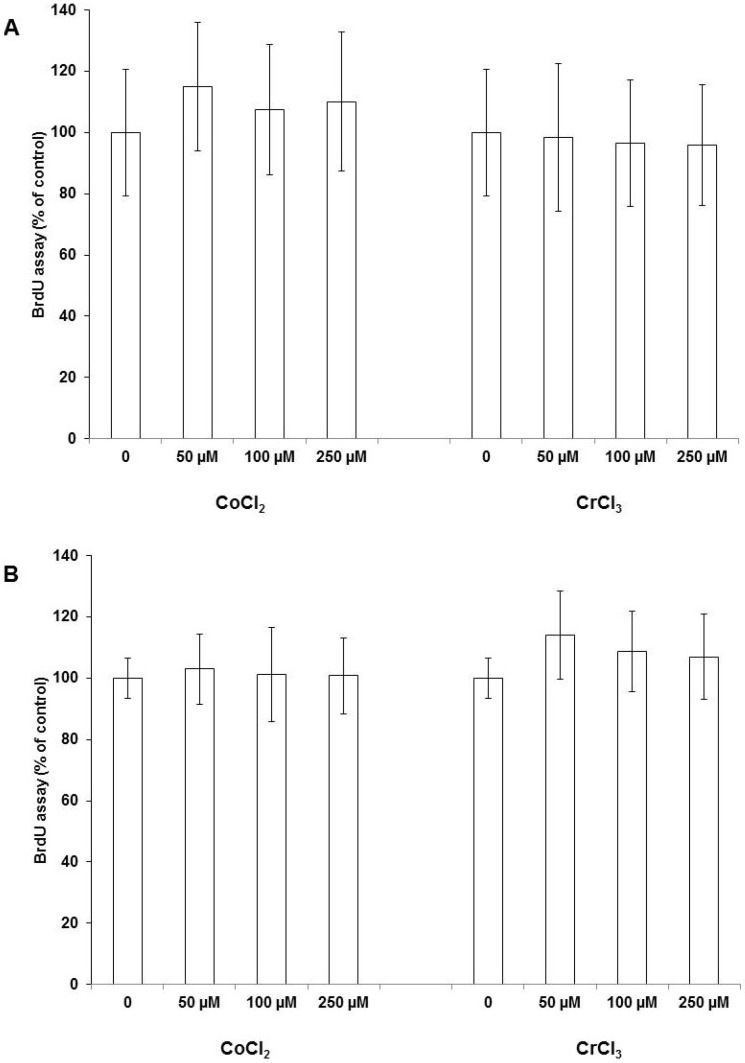
Proliferation activity of MG63 (**A**) and SaOs-2 (**B**) cells after 48 h stimulation with CoCl_2_ and CrCl_3_. Data represent the mean and standard deviation of four independent experiments, each performed in triplicate.

Proliferation of osteoblasts was determined by quantification of Bromodeoxyuridine (BrdU) incorporation. As shown in Figure 1, all Co^2+^ concentrations induced a slight, but not significant, increase in proliferation activity for MG63 as well as SaOs-2 cells.

Additionally, for Cr^3+^ treatment, no effects on cell proliferation were observed for any ion concentration. To further validate this result, we investigated the expression of the proliferation marker proliferating cell nuclear antigen (PCNA) using quantitative RT-PCR in MG63 cells. We observed no change in PCNA expression at a sub-confluent concentration of 1 × 10^5^ cells upon stimulation with Co^2+^ in a concentration range between 0–250 µM 

### 2.2. Cell Migration

**Figure 2:**
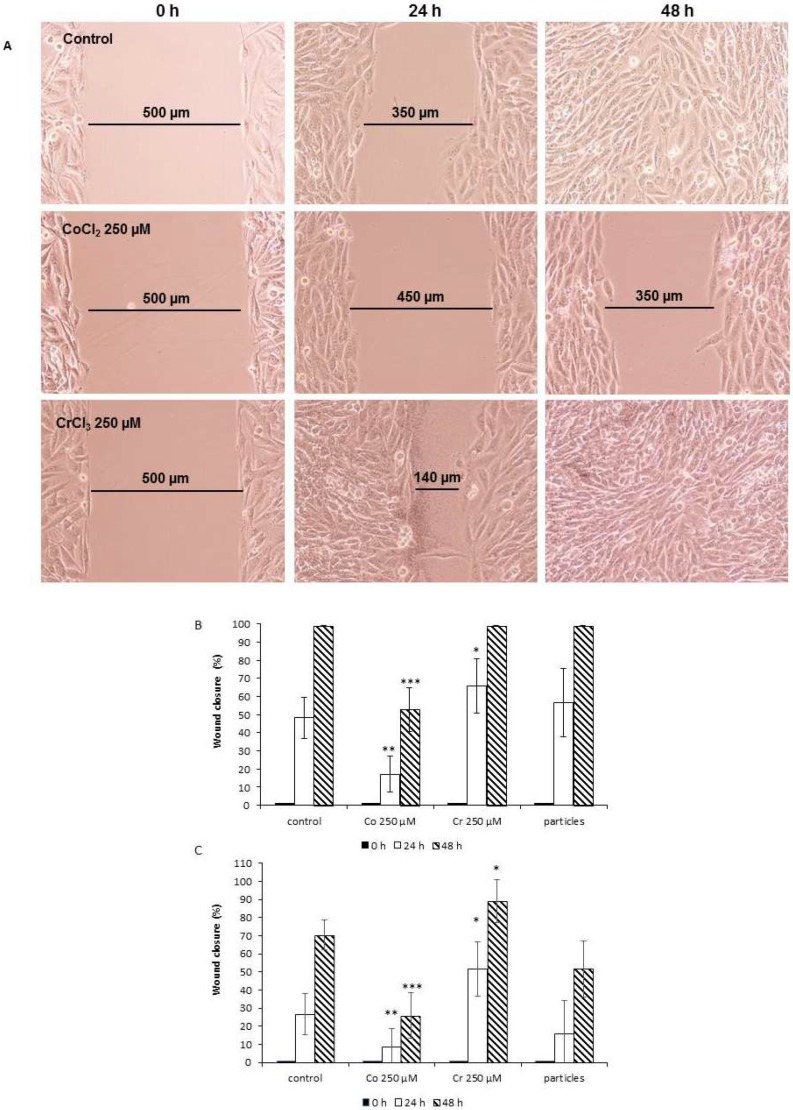
Effect of treatment with CoCl_2_, CrCl_3_, and CoCr particles on relative wound closure in MG63 and SaOs2 cells, measured using ibidi^®^ silicone inserts in an in vitro wound healing assay over 48 h. (**A**) Representative images of MG63 cells. Summary of the wound healing assay results for (**B**) MG63 and (**C**) SaOs-2 cells. Data represent the mean of four independent experiments. Data were examined in the time course using ANOVA (MG63: Co^2+^ *p* < 0.0001, Cr^3+^ *p* < 0.05, CoCr particles not significant; SaOs-2: Co^2+^ *p* < 0.001, Cr^3+^ *p* < 0.01, CoCr particles *p* < 0.01). For comparison of paired samples of treated cells vs. untreated control the post-hoc Bonferroni test was used (* *p* < 0.05, ** *p* < 0.01, *** *p* < 0.001).

The influence of Co^2+^ and Cr^3+^ ions and CoCr particles on the migration capability of MG63 and SaOs-2 cells was analyzed using a wound healing assay. In MG63 cells, the standardized gap was reduced by 50 ± 11.3%, and after 48 h, a complete closure of the gap was seen. Co^2+^ treatment of cells resulted in a deceleration of this process, even after 48 h, the gap was still open. In contrast, Cr^3+^ accelerated this process in comparison to the unstimulated control. After 24 h, the gap was reduced by 67.0 ± 15.1% and completely closed after 48 h. Representative images for Co^2+^ and Cr^3+^ treated MG63 cells are shown in Figure 2A. The treatment of cells with CoCr particles (1 × 10^6^/well) had no significant effect. The summary of the wound healing assay in MG63 and SaOs-2 cells are summarized in Figure 2B,C.

### 2.3. Expression of Osteogenic Markers

Human osteoblast-like MG63 cells were incubated without stimulus (control), and with CoCl_2_ (250 µM), CrCl_3_ (250 µM), and CoCr-particles (1 × 10^6^/well) in 12-well plates for 24 h.

Fifteen out of 84 genes were found to be expressed at a low level (*C*_t_ > 35 cycles), which was considered as non-detected. After normalization of the gene target expression to glyceraldehyde 3-phosphate dehydrogenase (GAPDH), 29 genes were found to be differentially regulated in CoCl_2_ treated MG63 cells compared to the untreated control by at least a factor of 2. The majority of genes were down-regulated (*n* = 25) (Table 1), whereas only four genes were found to be up-regulated (Table 2).

**Figure 3:**
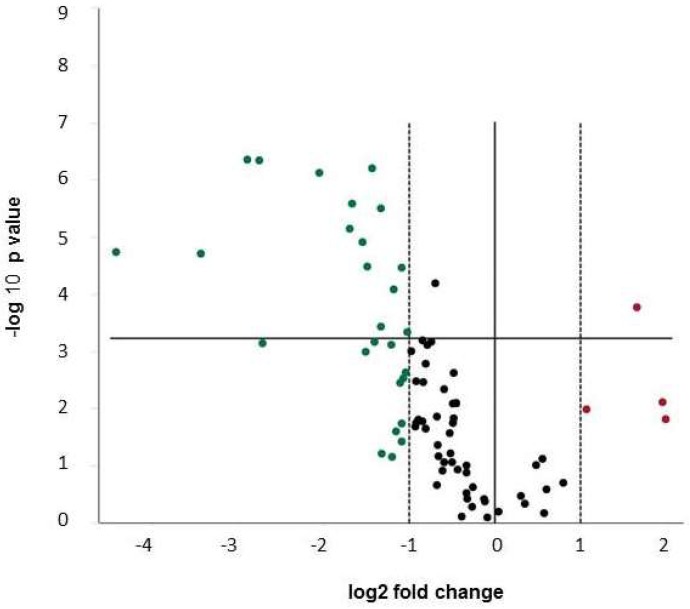
Volcano Plot for changes of gene expression in MG63 cells after 24 h incubation with CoCl_2_ vs. unstimulated control. Expression data are normalized vs. glyceraldehyde 3-phosphate dehydrogenase (GAPDH). The statistical significance is plotted versus fold-change on the *y*- and *x*-axes, respectively. The horizontal line represents the significance level (*p* = 0.0006). Up-regulated genes are depicted in red, down-regulated genes in green and genes regulated by less than factor 2 in black. Low abundant transcripts are included. Data are summarized from four independent experiments.

The volcano plot (Figure 3) reflects the predominant down-regulation of transcription of osteogenic markers.

The strongest effect was seen on the expression of different collagens e.g., collagen 14A1 (−6.5-fold down-regulation, *p* = 0.0007), Col15A1 (−6.7-fold down-regulation, *p* = 4.58 × 10^−7^), and Col3A1 (−10.8-fold down-regulation, *p* = 1.96 × 10^−5^), but also Col1A1 (−2.5-fold down-regulation, *p* = 0.00037) and Col1A2 (−3.2-fold down-regulation, *p* = 7.20 × 10^−5^) as well as other extracellular matrix proteins, such as COMP (−2.65-fold down-regulation, *p* = 0.00068) and biglycan (−2.1-fold down-regulation, *p* = 3.45 × 10^−5^). Interestingly, different cytokines also involved in the regulation of bone homeostasis were decreased in expression e.g., members of the TGF-β family (TGF-β2 −7.4-fold, *p* = 4.5 × 10^−5^ and TGF-β3 −2.3-fold, *p* = 8.3 × 10^−5^) and BMPs (BMP4 −2.8-fold, *p* = 3.3 × 10^−5^ BMP5 −21.5-fold, *p* = 1.8 × 10^−5^). Co^2+^ ions also down-regulated the expression of downstream targets of the TGF-β family signaling cascade SMAD1 (−2.9-fold) and SMAD2 (−2.1-fold).

MG63 cells treated with Cr^3+^ did not show a significant change in the expression in any of the 84 genes tested.

**Figure 4:**
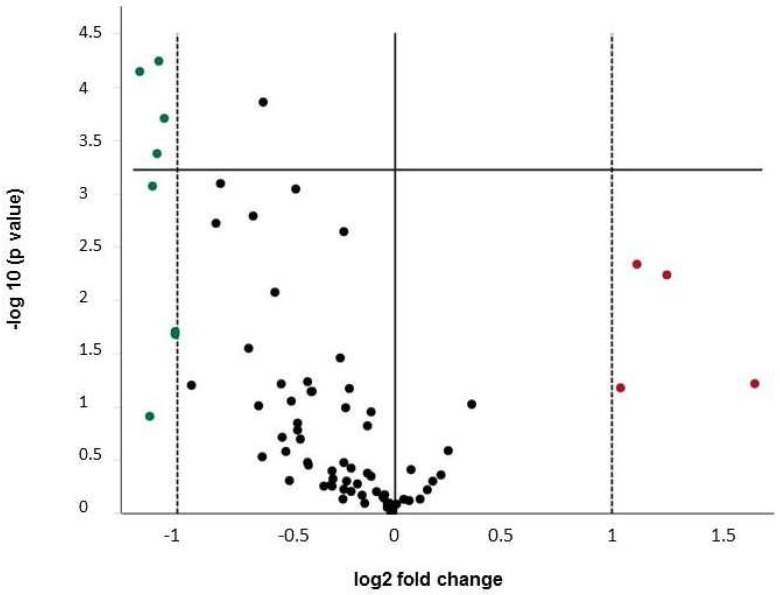
Volcano Plot for changes of gene expression in MG63 cells after 24 h incubation with Co/Cr particles vs. unstimulated control. Expression data are normalized to GAPDH. The statistical significance is plotted versus fold-change on the *y*- and *x*-axes, respectively. The horizontal line represents the significance level (*p* = 0.0006). Up-regulated genes are depicted in red, down-regulated genes in green and genes regulated by less than factor 2 in black. Data are summarized from four independent experiments.

CoCr particles at a concentration of 1 × 10^6^/well (3.83 cm^2^) affected the gene expression of several genes by at least a factor of 2 (Figure 4, Table 3 and Table 4), eight genes were down-regulated, four genes up-regulated. Interestingly, down-regulation of Col14A1 (−2.01-fold) and Col15A1 (−2.1-fold) was also seen, along with down-regulation of Co^2+^ treated cells, although to a lesser extent. Additionally, the down-regulation of BMP5 (−2.2-fold) was reproducible in particle treated cells.

### 2.4. Collagen Staining with Sirius Red

**Figure 5:**
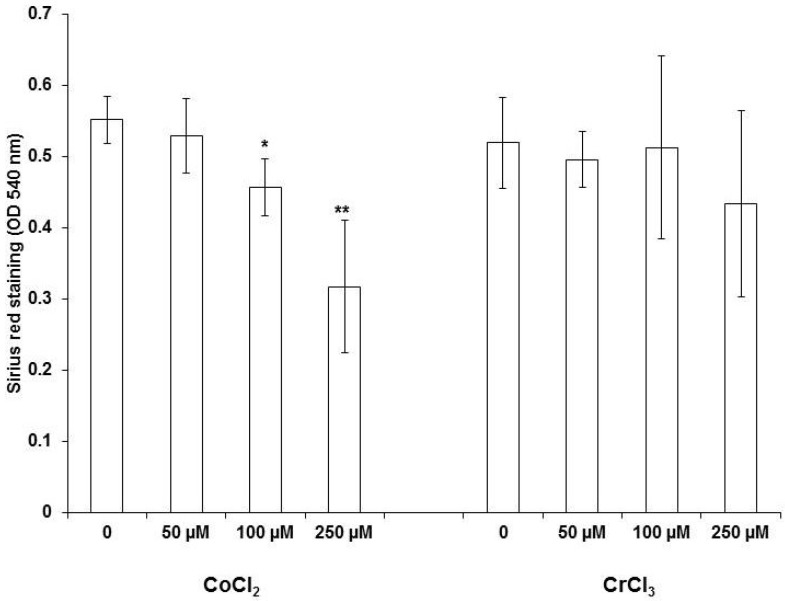
Influence of Co^2+^ and Cr^3+^ on collagen secretion by MG63 cells determined using Sirius red staining, one-way ANOVA *p* < 0.001 for CoCl_2_ treatment. For pairwise comparison a post hoc Bonferroni test was applied (* *p* < 0.05, ** *p* < 0.01 vs. unstimulated control). Data represent results of four independent experiments, each performed in triplicate.

In line with the array data, we found that CoCl_2_ reduced the total collagen production significantly in MG63 cells as shown by Sirius red staining. Figure 5 shows the dose-dependent decrease in collagen release upon Co^2+^ ion stimulation. As expected, no effect on collagen production was found in Cr^3+^ treated cells.

## 3. Discussion

Abrasive wear particles from endoprosthetic implants are known to induce adverse reactions in periprosthetic tissues (ARMD) such as osteolysis [24,25]. It has been shown that ARMD is associated with elevated serum levels of cobalt and chromium [25]. Cobalt measurements in the synovial fluid of failed metal on metal (M-o-M) hip replacements revealed a cobalt concentration of up to 30 µM [26]. Furthermore, a median concentration of 6.4 µg/g cobalt (range: 0.2–262 µg) within periprosthetic tissue was found around failed M-o-M hip implants [27]. For this reason, the Co^2+^ and Cr^3+^ ion concentrations were chosen in this manuscript. Co^2+^ and Cr^3+^ have been shown to modulate the expression of cytokines and chemokines in bone cells, inducing osteolytic processes or changes in osteoblast migration along chemokine gradients [9]. Interestingly, we have shown before that the C-X-C chemokine receptor type 4 (CXCR4) is up-regulated by different types of CoCr particles as well as dose-dependently by soluble Co^2+^ ions dose-dependently in osteoblast-like cells (MG63 and SaOs-2) [28,29]. CXCR4 is known to regulate osteoblast migration along chemokine gradients [30]. It has been shown that members of the TGF-β family are important cytokines involved in the maturation of osteoclasts and osteocytes [31], as well as regulation of bone formation by regulating extra cellular matrix deposition. In a previous study, we analyzed the influence of Co^2+^ and Cr^3+^ on the expression of the three TGF-β isoforms. After the binding of TGF-β to its cell surface, receptor Smad2 and Smad3 are phosphorylated and form complexes with Smad4 [32]. These complexes translocate to the nucleus and activate target genes including those required for assembly of the collagen extracellular matrix [32,33]. Our previous data show that bivalent cobalt ions and trivalent chromium ions have different effects on bone forming cells. While Co^2+^ down-regulated the expression of all three TGF-β isoforms in osteoblast-like cells, no inhibitory effect on mineralization was seen in the tested concentrations. Cr^3+^, however, did not influence the expression of TGF-β but strongly inhibited the mineralization in vitro [23]. These data suggest that the influence of Co^2+^ ions on bone homeostasis may be related to the inhibitory effect on the transcription of the bone formation regulating cytokines e.g., TGF-β1-3 and thereby on the bone forming activity of osteoblasts e.g., collagen production. The level of mineralization is determined by the expression of genes such as collagen, however, other post-transcriptional factors seem to have a stronger effect on mineralization.

One possible explanation for this effect might be that CoCl_2_ initiates HIF-1α signaling [34]. HIF-1α has been shown to regulate fibrotic tissue changes by inducing collagen expression, as well as TGF-β signaling [35,36].

Cr^3+^ ions, however, did not influence the gene expression and therefore most likely interact with the mineralization process directly. Shah et al. showed comparable effects of cobalt and chromium on the mineralization of murine “osteoblast to osteocyte-like” cell line MLO-A5 [37]. The inhibition of mineralization by soluble Cr^3+^ or on soluble CrPO_4_ might be due to the binding of phosphate to Cr^3+^, thereby reducing the availability of the ionic form [23]. However, this interaction needs to be analyzed in further studies.

To further investigate the underlying mechanisms we performed the RT^2^ human osteogenesis PCR Profiler Array covering 84 genes involved in osteogenesis. Twenty-nine genes were found regulated in CoCl_2_ treated MG63 cells compared to the untreated control by at least a factor of 2. These present data strengthen our recently published findings of a down-regulation of all TGF-β isoforms by Co^2+^, with the strongest effect on the expression of TGF-β2. Additionally, we observed that the expression of two other members of the TGF-β superfamily, BMP4 and BMP5, was significantly down-regulated by Co^2+^. Interestingly, BMP5 has been shown to up-regulate osteoclast formation, thereby inducing increased bone loss [38]. In contrast to Co^2+^/Cr^3+^-ions, CoCr particles showed no significant effect on migration and only a moderate effect on gene expression. There might be an effect after a prolonged exposure of osteoblasts to metal particles as a result of the intracellular corrosion, however, this was not investigated in this study.

The current study gives deeper insights into the mechanisms of how divalent Co^2+^ and trivalent Cr^3+^ alter bone homeostasis and contribute to osteolysis. Experiments using osteoclasts were beyond the scope of the study—nevertheless bone resorption caused by osteoclasts is a very important topic in bone homeostasis. Beside the canonical osteoclasts differentiation by receptor activator of NF-κB–receptor activator of NF-κB ligand (RANK–RANKL) interaction, Sabokbar et al. described a non-canonical, RANKL independent way of osteoclast differentiation [39]. The migration experiments showed that Co^2+^ (250 µM) decelerated migration whereas Cr^3+^ at the same concentration accelerates the migration rate compared to untreated MG63 cells. One reason for this effect could be changes in proliferation or metabolic activity. As shown previously the metabolic activity was slightly up-regulated by Co^2+^, whereas Cr^3+^ exhibited no effects on the cell metabolisms measured by water soluble tetrazolium (WST)-1 test [23]. Since metabolic activity determined using the WST-1 assay cannot be equated with proliferation, the proliferative activity of MG63 and SaOs-2 cells was measured after 48 h by determination of DNA replication using the BrdU assay—the results are similar to those of the WST-1 data, as well as the quantitative RT-PCR for the proliferation marker PCNA. These results are in agreement with the findings of other groups. Li and Wang reported that migration of smooth muscle cells is inhibited by Co^2+^ ions, whereas cell proliferation was only slightly increased [40].

As changes in proliferation of osteoblasts could not explain the decreased migratory capacity upon Co^2+^ ion stimulation, other pathways must be involved. The deposition of the extracellular matrix is important for cell migration. The extracellular matrix influences cellular migration, besides acting as a major reservoir of releasable chemokines it also provides guidance and confinement of the cell body resulting in a shape adaptation in order to move [41]. Therefore, the observed down-regulation of various extracellular matrix molecules, e.g., collagens and biglycan, could explain the reduced migratory capacity. The down-regulation of different collagen genes is in agreement with our observation that the production of total collagen is significantly reduced by CoCl_2_. Interestingly, TGF-β has been shown to play a crucial role in the expression regulation of various collagens. TGF-β/Smad3 stimulation favored the secretion of collagen-3 versus collagen-1 [42]. A critical role for Col3 in skeletal development is suggested by its appearance in mesenchymal condensations preceding cartilage and bone formation [43], its requirement for growth acceleration of osteoblasts [44] thus making a role of Col3 in fracture healing or ingrowth of prosthesis possible [45].

Interestingly, TGF-β 1 significantly up-regulated collagen type XIV expression [46]. These changes in ECM production could explain the reduced migratory capacity of MG63 and SaOs-2 cells after incubation with Co^2+^ ions.

We are aware that our study is limited by several factors. The use of the osteogenic array for the analysis of the effects of Co^2+^ and Cr^3+^ on gene expression limits our findings to genes which are involved in functions of osteoblasts. As shown earlier, other genes are also affected [28,29]. Furthermore, instead of primary osteoblasts which exhibit large inter-individual variability as well as limited cell division, we used osteoblast-like cell lines for our experiments. MG63 and SaOs-2 cells do not have the limitations of primary cells but their tumor derived origin must be taken into account. The concentrations of Co^2+^ and Cr^3+^ used in this study are within a range described in literature [9] e.g., Mabilleau et al. used concentrations up to 100 µM and observed no cytotoxic effects [47], some other authors worked with even higher concentrations up to 370 µM for Co^2+^ and 2.8 mM for Cr^3+^ [48,49]. In previous studies, we tested all three concentrations and found no adverse effects in PCR experiments [23].

Besides contributing to the understanding of osteoblast functional pathways, our data contribute to the knowledge of changes in gene expression patterns induced by metallic implants and/or their corrosion products. These findings may also have a clinical impact and may support the development of so called “smart surfaces” that may recognize pathological conditions e.g., osteolysis in periprosthetic tissues or bacterial colonization on implant surfaces at early stages. Rebound strategies may be then introduced by eluting for example anti-inflammatory or antimicrobial drugs. All these interesting topics should be the focus of further studies.

## 4. Material and Methods

### 4.1. Cell Culture

MG63 and SaOs-2 osteoblast-like cells were cultured in Dulbecco’s modified eagle medium (DMEM), supplemented with 10% fetal calf serum (FCS), 1% penicillin/streptomycin at 37 °C, and in 5% CO_2_ in a humidified atmosphere. For all experiments the cells were used from the 3th to the 7th passage.

Cell culture experiments for cell migration, RT^2^ Profiler PCR Array, and experiments for collagen determination were performed in 12-well cell culture plates (growing area: 3.83 cm^2^, final volume: 2 mL). For the determination of proliferation, 5 × 10^3^ cells were seeded into 96-well plates (growing area: 0.32 cm^2^, final volume 100 µL). After overnight adherence of the cells, the medium was changed and the cells were treated with the given concentrations of cobalt(II)chloride and chromium(III)chloride, as well as with Co-35Ni-20Cr-10Mo particles. The MG63 and SaOs-2 cell lines are well-characterized [50,51]—they share properties with primary human osteoblasts. The response to parathyroid hormone is similar, as well as the inhibition of proliferation with 1,25-(OH)_2_D_3_, the 1,25-(OH)_2_D_3_ dependent increase of alkaline phosphatase activity and osteocalcin mRNA, and protein expression [52,53,54,55]. Therefore, MG63 and SaOs-2 cell lines can be considered as well-established models for osteoblast experiments and have been used in previous studies by us [28,29].

### 4.2. Preparation of CoCl_2_ and CrCl_3_ Solutions

For the application of Co^2+^ and Cr^3+^ ions in the cell culture experiments, 100 mM stock solutions were prepared. For the preparation of Co^2+^, CoCl_2_ (Sigma Aldrich, Taufkirchen, Germany) was dissolved in water. For the preparation of Cr^3+^, CrCl_3_ × 6 H_2_O was dissolved in water. When this takes place, CrCl_3_ × 6 H_2_O forms as in initial species trans-[Cr^3+^(H_2_O)_4_Cl_2_]Cl × 2 H_2_O which appears emerald green. Within a time period of two weeks, this complex is reorganized to the final species [Cr^3+^(H_2_O)_6_]Cl_3_ (purple)—this stable species was used for all chromium experiments.

### 4.3. Preparation of Particles

The wear particles of the Co-35Ni-20Cr-10Mo alloy (CoCr) were produced according to Buchhorn et al. [56] and as described previously [55]. Briefly, Co-35Ni-20Cr-10Mo containers were filled with bars of the same material and filled with absolute ethanol followed by eccentrical and continuous rotation at room temperature. By tumbling in this way, the bars rubbed against the wall of the container and generated the particles. The chemical composition and relative purity of the particles was validated by energy-dispersive X-ray (EDX) spectroscopy. The particle size spectra in the basic stock-suspension ranged from less than 0.1 µm to about 200 µm. More than 80% of all particles were smaller than 5 µm and the maximum size distribution was about 2 µm [57].

One milliliter of the metal suspension was applied on a suction filter using a 200 nm pore size polyester membrane (Steritech, Charlotte, NC, USA). The dried filters were applied to sterile glass vials containing 10 mL DMEM medium (without any supplements) and irradiated under UV-light overnight in a laminar flow. For the experiments, stock solutions of all particle types containing 1 × 10^8^ particles per mL were prepared—the highest concentration used in cell culture was 1 × 10^6^ particles/well. All particle solutions tested endotoxin free using the E-TOXATE test (Sigma-Aldrich, Taufkirchen, Germany).

### 4.4. Cell Viability

The viability of MG63 and SaOs-2 cells after stimulation with CoCl_2_ and CrCl_3_ was measured by Trypan blue exclusion test. In viable cells Trypan blue is not absorbed, however, it traverses the cells membranes in dead cells. Cells used for experiments exhibited a viability of >95%.

### 4.5. RT^2^ Profiler PCR Array (PAHS-026ZF)—Human Osteogenesis

The RT^2^ Profiler PCR Array “Human Osteogenesis” (Qiagen, Hilden, Germany) covers 84 genes involved in osteogenesis. The arrays were performed according to the manufacturer´s instructions. Human osteoblast-like MG63 cells were incubated without stimulus (control), and with CoCl_2_ (250 µM), CrCl_3_ (250 µM), and CoCr-particles (1 × 10^6^/well) in 12-well plates for 24 h. This experimental design was repeated in 4 independent experiments. RNA was extracted using the RNeasy kit with an on-column DNAse digestion (Qiagen). For cDNA synthesis with the RT^2^ First Strand Kit (Qiagen) 1 µg of total RNA was used. Briefly, following a genomic elimination step of 5 min incubation with Buffer GE at 42 °C, the reverse transcription was performed for 15 min at 42 °C. For the PCR reaction the cDNA reaction volume of 20 µL was mixed with water, and the 2× RT^2^ SYBR green mastermix. 25 µL of this mixture were consistently pipetted into all wells of the 96-well preloaded PCR plate. The PCR was performed on a Roche Light Cycler 480 (software 1.5.0 SP4) (Roche, Rotkreuz, Switzerland) according to protocol F of the manufacturer´s manual.

### 4.6. Cell Migration Assay

For the cell migration assays, 2-well silicone inserts with a defined free gap (ibidi^®^, Martinsried, Germany) were placed on 12-well cell culture plates. 70 µL of a cell suspension, containing 3 × 10^5^ MG63 or SaOs-2 cells/mL, were filled into the chambers and incubated overnight. After removal of the inserts, a cell free gap of 500 µm was visible under the light microscope. After addition of Co^2+^, Cr^3+^, and CoCr particles, the closure of the gap was analyzed over a period of 48 h.

### 4.7. Detection of Proliferation

The proliferation of MG63 and SaO-2 cells was measured using the BrdU cell Proliferation Assay Kit (Cell Signaling Technology, Frankfurt, Germany) according to the manufacturer´s instructions. This kit detects 5-bromo-2′-deoxyuridine (BrdU) incorporated into cellular DNA during cell proliferation using an anti-BrdU antibody. Briefly, 5 × 10^3^ cells were seeded into 96-well cell culture plates and stimulated with CoCl_2_ and CrCl_3_ (0–250 µM) for 48 h. The fixed and permeabilized cells were subsequently incubated with the anti-BrdU antibody and then with the horseradish peroxidase (HRP)-conjugated secondary antibody. Incubation with 3,3′,5,5′-tetramethylbenzidine (TMB) stained the cells blue—it’s intensity correlates with BrdU incorporation. After 30 min the reaction was stopped by adding sulphuric acid. The absorbance was measured at 450 nm using a Tecan Infinite F200 Pro multiplate reader (Tecan, Männedorf, Switzerland).

### 4.8. Detection of Collagen Secretion

For determination of collagen secretion, 3 × 10^5^ cells per well in a 12-well plate were incubated for 48 h with CoCl_2_ and CrCl_3_, respectively. Then the cells were washed three times with PBS, fixed for 30 min with formalin and washed three times with destilled water. 400 µL of Sirius Red staining solution (0.1% direct red 80 (Sigma, Taufkirchen, Germany) in water-saturated picric acid) was added to each well and incubated for 30 min. Unbound dye was removed by repeated washing with 2.5% acetic acid. To quantify collagen staining the dye was extracted with 0.1 N NaOH and the absorption was measured at 540 nm.

### 4.9. Statistics

The statistical analysis was performed using SPSS 24. If not stated otherwise data were expressed as mean ± standard deviation (SD). Data from the stimulation experiments were analyzed using the one-way analysis of Variance (ANOVA) with different metal concentrations or with different time points as independent factors, and with post hoc Bonferroni test for pairwise analysis.

### 4.10. Analysis of Array Data

For analysis of RT^2^ Profiler PCR array data, a web-based software (available online: https://www.qiagen.com/de/shop/genes-and-pathways/data-analysis-center) was used.

The ΔΔ*C*_t_ method was used to calculate fold changes between treated groups and control group in which delta *C*_t_ is calculated between gene of interest and GAPDH as a housekeeper. The statistical significance of differences among the treated groups vs. untreated control was examined by Student’s *t* test. As a result of multiple testing the *p* value for significance was adjusted to 0.0006.

## Figures and Tables

**Table 1:** Genes down-regulated by CoCl_2_ after 24 h by at least a factor of 2 (normalization of *C*_t_-values to glyceraldehyde 3-phosphate dehydrogenase (GAPDH)). The significance level was adjusted for multiple testing to 0.0006 (bold *p*-values are significant). A = in either the control or the test sample, the average *C*_t_ is high (>30 cycles).

Gene Symbol	Fold Regulation	Comments	*p*-Value
*BGN*	−2.12		**3.45254 × 10^−5^**
*BMP4*	−2.81		**3.30439 × 10^−5^**
*BMP5*	−21.52	A	**1.83701 × 10^−5^**
*BMPR2*	−2.15		0.003576291
*CDH11*	−4.14		**7.55543 × 10^−7^**
*CHRD*	−2.22		0.025290803
*COL14A1*	−6.54		0.000723157
*COL15A1*	−6.74	A	**4.58304 × 10^−7^**
*COL1A1*	−2.52		**0.000370615**
*COL1A2*	−3.23		**7.20666 × 10^−6^**
*COL3A1*	−10.78		**1.96377 × 10^−5^**
*COL5A1*	−2.03		0.000462367
*COMP*	−2.65		0.000687369
*IGF1R*	−2.85		0.001021934
*ITGA1*	−2.13		0.037993599
*ITGA3*	−2.12		0.018352525
*MMP2*	−2.7		**6.30662 × 10^−7^**
*RUNX2*	−2.51		**3.16733 × 10^−6^**
*SMAD1*	−2.93		**1.23015 × 10^−5^**
*SMAD2*	−2.06		0.002343501
*SOX9*	−3.18		**2.62519 × 10^−6^**
*TGFB2*	−7.4	A	**4.4503 × 10^−7^**
*TGFB3*	−2.27		**8.28135 × 10^−5^**
*TWIST1*	−2.32		0.00077027
*VEGFB*	−2.1		0.002973727

**Table 2:** Genes up-regulated by CoCl_2_ after 24 h by at least a factor of 2 (normalization of *C*_t_-values to GAPDH). The significance level was adjusted for multiple testing to 0.0006 (bold *p*-values are significant), A = in either the control or the test sample, the average *C*_t_ is high (>30 cycles).

Gene Symbol	Fold Regulation	Comments	*p*-Value
*BMP6*	4		0.015432431
*EGF*	3.89		0.00777753
*FGF1*	2.11		0.010384109
*PDGFA*	3.15	A	**0.000170489**

**Table 3:** Genes down-regulated after 24 h of stimulation with particles by at least a factor of 2 (normalization of *C*_t_-values to GAPDH). The significance level was adjusted for multiple testing to 0.0006 (bold *p*-values are significant), A = in either the control or the test sample, the average *C*_t_ is high (>30 cycles).

Gene Symbol	Fold Regulation	Comments	*p*-Value
*BMP5*	−2.16		0.0008533
*BMP6*	−2.01		0.0209783
*COL14A1*	−2.01		0.0197225
*COL15A1*	−2.12	A	**5.71 × 10^−5^**
*PDGFA*	−2.13		**0.0004219**
*SMAD1*	−2.25		**7.161 × 10^−5^**
*SMAD4*	−2.18		0.1233691
*VDR*	−2.08		**0.0001971**

**Table 4:** Genes up-regulated after 24 h of stimulation with particles by at least a factor of 2 (normalization of *C*_t_-values with GAPDH). At a significance level of 0.0006 (adjusted for multiple testing) no significant up-regulation was found, A = in either the control or the test sample, the average *C*_t_ is high (>30 cycles) B: the relative expression level is low (*C*_t_ > 30, in both control and test samples).

Gene Symbol	Fold Regulation	Comments	*p*-Value
*FGF1*	2.06	B	0.0666043
*GDF10*	3.15	B	0.0609019
*NOG*	2.39	A	0.0058095
*VEGFA*	2.17		0.0046092

## Abbreviations

ANOVA	Analysis of Variance
ARMD	Adverse Reactions on Metallic wear Debris
BMP	Bone Marrow Protein
BrdU	Bromodeoxyuridine
COL	Collagen
COMP	Cartilage Oligomeric Matrix Protein
CXCR4	C-X-C chemokine Receptor type 4
DMEM	Dulbecco’s Modified Eagle Medium
ECM	Extracellular Matrix
EDX	Energy Dispersive X-ray spectroscopy
FCS	Fetal Calf Serum
GAPDH	Glyceraldehyde 3-phosphate dehydrogenase
HIF	Hypoxia-Inducible Factor(s)
HRP	Horseradish peroxidase
M-o-M	Metal-on-metal
PBS	Phosphate Buffered Saline
PCNA	Proliferating Cell Nuclear Antigen
PCR	Polymerase Chain Reaction
RANK	Receptor Activator of NF-κB
RANKL	Receptor Activator of NF-κB Ligand
RT-PCR	Real-Time PCR
SMAD	related to “MAD”(mothers against decapentaplegic) of D. Melanogaster and “Sma”(small body size) of C. elegans
TGF	Transforming Growth Factor
THA	Total Hip Arthroplasty
WST	Water Soluble Tetrazolium
